# Supplementary figures and images for: Data reuse and the open data citation advantage
Source: PeerJ. 2013 Oct 1;1:e175. doi: 10.7717/peerj.175 (PMC3792178; doi:10.7717/peerj.175)

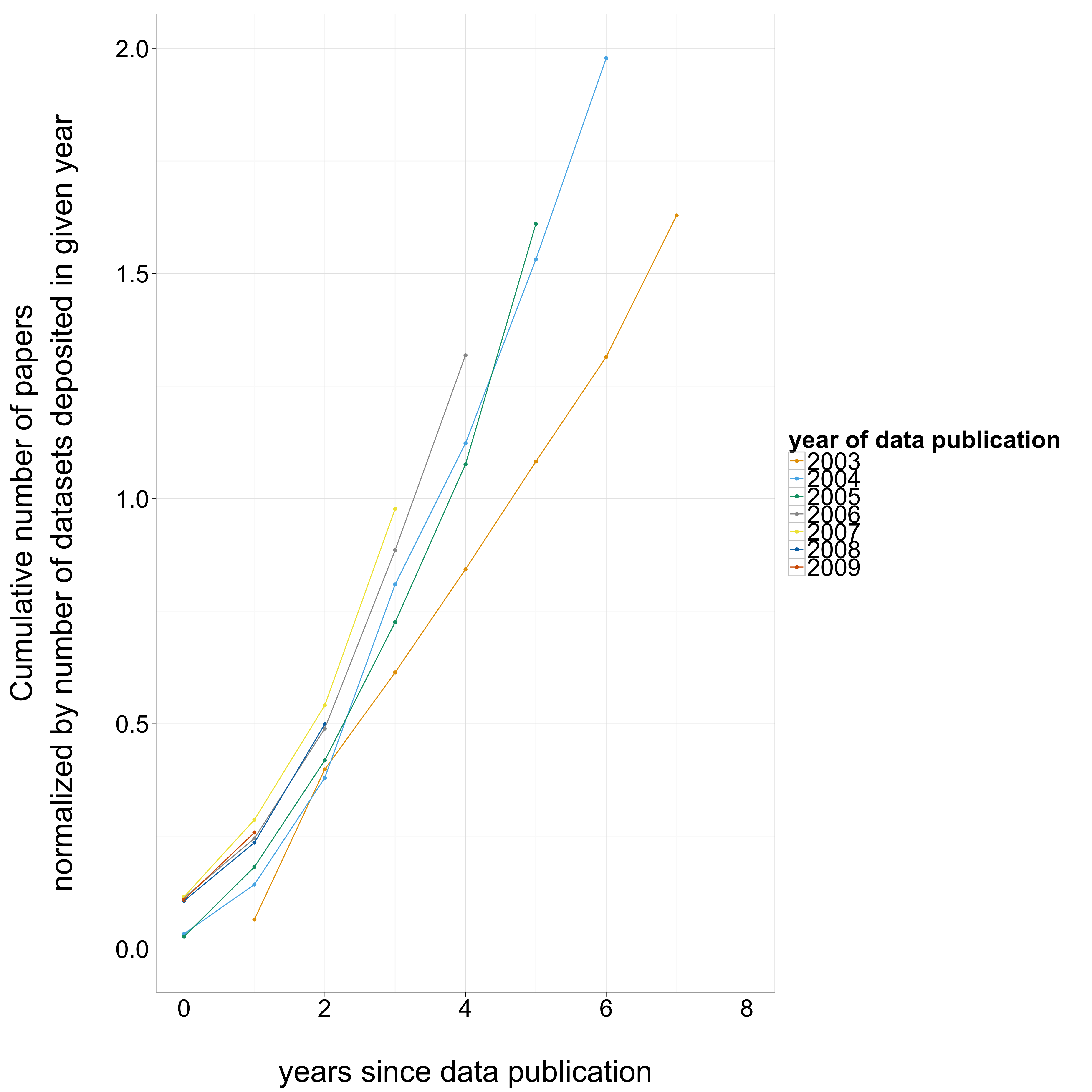

Supplement: Article S1 [file peerj-01-175-s001.png]
