# Supplementary material for: Data reuse and the open data citation advantage
Source: PeerJ. 2013 Oct 1;1:e175. doi: 10.7717/peerj.175 (PMC3792178; doi:10.7717/peerj.175)
Supplement: Figure S1 [file peerj-01-175-s002.pdf]

Supplemental Article S1

Validating the automated method of detecting data availability

Our method of identifying which articles create gene expression microarray data made a nontrivial number of errors: about 10% of the articles it identified as creating gene expression microarray data do not in fact create gene expression datasets.

The papers that are erroneously included in our subset to not create gene expression data, so they certainly don't have associated archived datasets: all erroneously included papers were automatically classified in the "no archived data" group.

If it were true that these erroneously-included articles received many more or many fewer citations than other articles in the group, their inclusion could influence the findings of this study. To verify our assumption that the influence of these mistakenly-included articles is in fact small, we manually reviewed a random 226 of the 11k (get exact number) articles. Of these manually reviewed articles, 206 did indeed create gene expression microarray data, and 20 did not (but satisfied the boolean-search query for other reasons).

Examining the citations of the 20 articles that did not create gene expression data revealed that these studies were cited less often than those that did create data: a mean of 26 citations compared to a mean of 32 citations. The overall distribution of citations for articles that did not create gene expression data is closer to zero than the distribution of citations for articles that did create gene expression data.

We took steps to verify our assumption that the influence of articles erroneously identified these mistakenly-included articles is in fact small. We began by manually reviewed a random 226 of the 11k (get exact number) articles to identify those which we were assuming had created gene expression microarray data but in fact had not.

We compared the distribution of those with errors to those without, calculated whether they were statistically different, and ran a regression with the known-correct sample only.

```
nCitedBy    N=226, 4 Missing

+---------+--------------------------+---+--------+
|         |                          |  N|nCitedBy|
+---------+--------------------------+---+--------+
|isCreated|    created-microarray-data|206|   31.86|
|         |created-microarray-data-not| 20|   26.30|
+---------+--------------------------+---+--------+
|  Overall|                          |226|   31.37|
+---------+--------------------------+---+--------+
```

This difference, however, was found to be not statistically significantly different at the $p<0.05$ level, using either a t-test on the log of the citation counts or a Wilcoxon rank sum test on the raw citation counts.

```
print(ttest_citedby)
    Welch Two Sample t-test

data:  nCitedBy by isCreated
t = 0.5747, df = 22.61, p-value = 0.5712
alternative hypothesis: true difference in means is not equal to 0
95 percent confidence interval:
 -14.47  25.59
sample estimates:
    mean in group created-microarray-data
                                    31.86
mean in group created-microarray-data-not
                                    26.30

print(ttest_log_citedby)
    Welch Two Sample t-test

data:  log(1 + nCitedBy) by isCreated
t = 1.331, df = 21.77, p-value = 0.1968
alternative hypothesis: true difference in means is not equal to 0
95 percent confidence interval:
 -0.2003  0.9175
sample estimates:
    mean in group created-microarray-data
                                    2.991
mean in group created-microarray-data-not
                                    2.632

print(wilcox_citedby)
    Wilcoxon rank sum test with continuity correction

data:  nCitedBy by isCreated
W = 2440, p-value = 0.1733
alternative hypothesis: true location shift is not equal to 0
```

To confirm that the erroneously-included articles were not driving the findings about the citation relationship with data availability, we ran a multivariate regression analysis on the subsample of 206 articles that we manually determined did in fact generate gene expression microarray data. The estimated effect is statistically significant and similar to the findings from the whole sample.

```
gfm_table(anova(annotated_merged_created))
|                                          | Df     | Sum Sq | Mean Sq | F value | Pr(>F) |
|------------------------------------------|--------|--------|---------|---------|--------|
| rcs(pubmed.date.in.pubmed, 3)            | 2.00   | 83.82  | 41.91   | 73.91   | 0.00   |
| rcs(journal.impact.factor.tr, 3)         | 2.00   | 18.69  | 9.35    | 16.48   | 0.00   |
| rcs(num.authors.tr, 3)                   | 2.00   | 4.03   | 2.01    | 3.55    | 0.03   |
| rcs(last.author.num.prev.pmc.cites.tr, 3) | 2.00   | 4.79   | 2.40    | 4.22    | 0.02   |
| factor(country.usa)                      | 1.00   | 0.05   | 0.05    | 0.09    | 0.77   |
| factor(dataset.in.geo.or.ae)             | 1.00   | 5.68   | 5.68    | 10.03   | 0.00   |
| Residuals                                | 177.00 | 100.37 | 0.57    |         |        |
calcCI.exp(annotated_merged_created, "factor(dataset.in.geo.or.ae).L")
                                  param  est ciLow ciHigh     p
Estimate factor(dataset.in.geo.or.ae).L 1.32  1.11   1.57 0.002
```
